# Synthesis and characterization of fluorescence-labelled silica core-shell and noble metal-decorated ceria nanoparticles

**DOI:** 10.3762/bjnano.5.251

**Published:** 2014-12-16

**Authors:** Rudolf Herrmann, Markus Rennhak, Armin Reller

**Affiliations:** 1 Institut für Physik, Universität Augsburg, Universitätsstr. 1, D-86159 Augsburg, Germany

**Keywords:** fluorescence labelling, noble metal nanoparticles, platinum-decorated ceria nanoparticles, perylene diimide, polyorganosiloxane core, silica core-shell nanoparticles

## Abstract

The present review article covers work done in the cluster NPBIOMEM in the DFG priority programme SPP 1313 and focuses on synthesis and characterization of fluorescent silica and ceria nanoparticles. Synthetic methods for labelling of silica and polyorganosiloxane/silica core–shell nanoparticles with perylenediimide derivatives are described, as well as the modification of the shell with thiol groups. Photometric methods for the determination of the number of thiol groups and an estimate for the number of fluorescent molecules per nanoparticles, including a scattering correction, have been developed. Ceria nanoparticles decorated with noble metals (Pt, Pd, Rh) are models for the decomposition products of automobile catalytic converters which appear in the exhaust gases and finally interact with biological systems including humans. The control of the degree of agglomeration of small ceria nanoparticles is the basis for their synthesis. Almost monodisperse agglomerates (40 ± 4–260 ± 40 nm diameter) can be prepared and decorated with noble metal nanoparticles (2–5 nm diameter). Fluorescence labelling with ATTO 647N gave the model particles which are now under biophysical investigation.

## Review

Within the general goal of the DFG priority programme SPP 1313, to study the unintended exposure of intended nanoparticles to biological systems, we decided to focus our research on oxidic nanoparticles (NP) applied technically in large scale, in particular silica, ceria, titania and zinc oxide. This review article concentrates on synthesis and characterization of fluorescent silica and ceria NP, the latter also decorated with noble metals as models for decomposition products of automobile catalysts. We have recently reported on fluorescence-labelled coated titania NP and their interaction with human cell lines [1] and pointed out that the determination of the biological effects of zinc oxide NP is problematic since they are sensitive towards phosphate ions [2]. This work will not be included in this article.

### The fluorescence dyes and the labelling process

**Figure 1:**
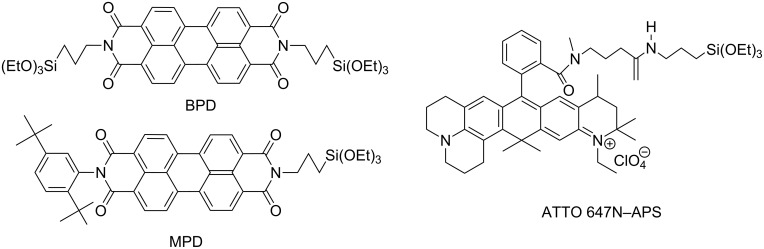
Fluorescent dyes used for labelling.

**Figure 2:**
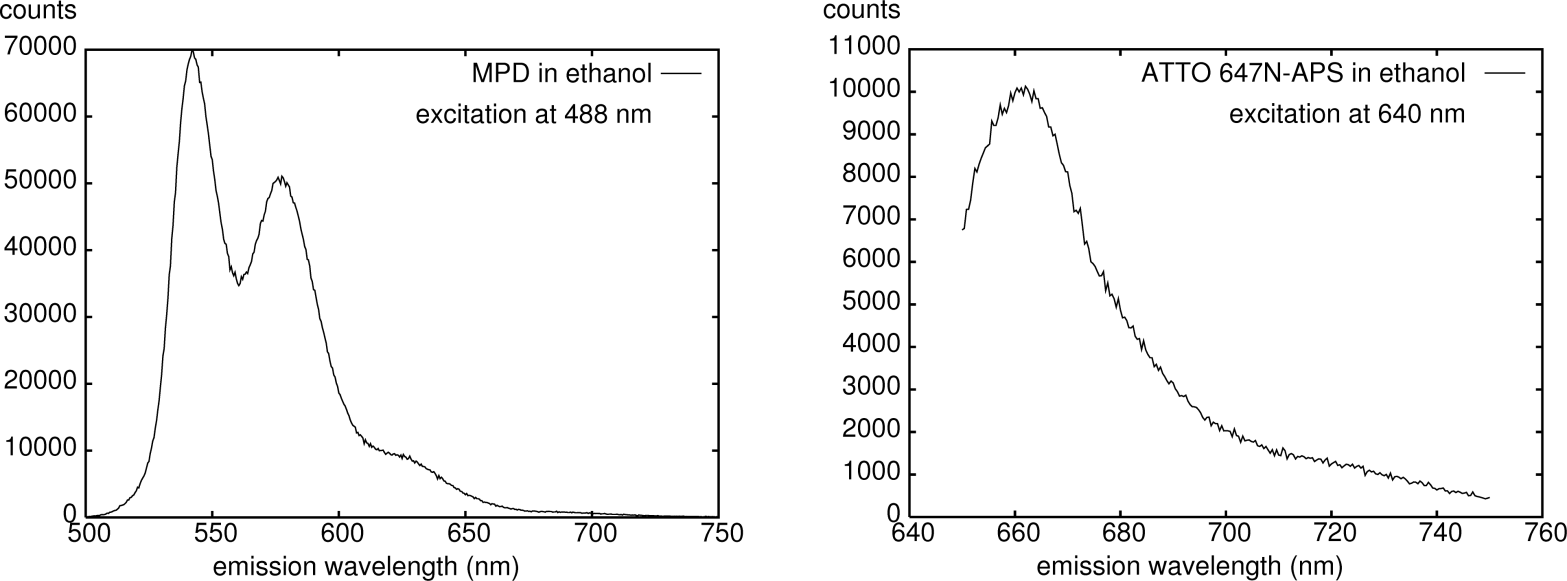
Fluorescence emission spectra in ethanol of MPD (excitation 488 nm, left) and ATTO 647N-APS (excitation 640 nm, right).

As principal means of investigation by our physicochemical and medicinal partners, confocal microscopy and other fluorescence-based methods were envisaged. Consequently, a proper choice of the fluorescent label is crucial. We first experimented with commercial dyes Cy3 and Cy5 having an emission in a suitable frequency range, but they turned out to be not sufficiently photostable under the experimental condititons. We then switched to perylenediimide derivatives which are known to be chemically and photochemically quite inert [3–4]. We prepared the dyes MPD (asymmetric) and BPD (symmetric) shown in Figure 1 containing triethoxysilyl groups to ensure easy connections to hydroxy groups at the surface of oxidic nanoparticles (MPD: [5]; BPD: [6–7]). A similar asymmetric dye is reported in [8]. The fluorescence emission spectrum of MPD in ethanol (Figure 2, left) upon excitation at 488 nm shows an intense peak at 540 nm (to be followed by fluorescence microscopy), a secondary peak at 575 nm, and a shoulder at 630 nm, together with a tail down to ca. 700 nm. Both MPD and BPD could be attached to Stöber type [9] silica NP [5] which were successfully applied in biological investigations [10–11], as well as to coated titania [1] and zinc oxide nanoparticles [2].

For experiments where several cell organelles are stained and detected in different ranges of fluorescence emission (channels), dyes with single sharp emission peaks are desirable. We therefore changed the dye for our experiments with ceria NP to ATTO 647N [12] which we applied as amide with (3-aminopropyl)triethoxysilane (Figure 1). This compound was obtained from commercial ATTO 647N NHS ester by reaction with (3-aminopropyl)triethoxysilane (APS) in DMF/ethanol. Upon excitation at 640 nm the dye shows a single strong emission around 660 nm which is confined to the red channel, avoiding any interference with, e.g., membrane stains emitting in the blue or green region (Figure 2, right). However, the chemical stability of ATTO 647N-APS is much lower than that of the perylene diimide-derived dyes. We found that its fluorescence is maintained reasonably well on storing in the dark in ethanol (intensity loss ≈0.1% per day) but appreciably less in water (approximately 1.5% per day), and similarily in cell media. Decomposition increases when exposed to light upon storage. There is no problem, though, with experiments in cell media not exceeding one weak of duration. At 120 °C at ceria NP the dye is completely destroyed within 90 min, the typical conditions for sterilization prior to biological experiments.

Oxidic nanoparticles like SiO_2_, TiO_2_, CeO_2_, ZnO and Al_2_O_3_ generally contain hydroxy groups at their surface to saturate dangling bonds at the margin of the three-dimensional networks. These groups can react with the triethoxysilyl group of APS-modified dyes and connect them covalently to the surface. In principle, each −Si(OEt)_3_ group can form three M–O–Si bonds, provided that the density of the hydroxy groups at the surface is sufficiently high to allow for unstrained bond lengths and angles. Where this is not the case and only one or two M–O–Si bonds are formed by the −Si(OEt)_3_ groups, one would a priori expect that this will result in increased sensitivity towards hydrolysis in aqueous media, leading finally to the removal of the fluorescence label from the NP [13]. However, we could detect detached dye only after storage of several months in water. There is no change at all on storage in ethanol. Labelling by the APS-derived perylene dyes is possible directly during the synthesis process (for SiO_2_, CeO_2_, and ZnO) by simply adding MPD or BPD to the reaction mixtures. For TiO_2_ and Al_2_O_3_ NP, one can apply MPD post-synthetically in ethanol solution at 140 °C in a closed vessel. Labelling with ATTO 647N-APS was generally done post-synthetically at 120 °C; the lower temperature was necessary due to the limited stability of the dye. Consequently, the number of dye molecules per NP remained lower than in the case of MPD.

In principle, many functional groups can be attached to the silica surface by constructing suitable derivatives with triethoxysilyl groups, e.g., APS itself [14] or various fluorescent molecules [15–20] including iridium complexes [21]. The presence of amino groups after the attachment of APS to silica can be used for an alternative approach to NP similar to our MPD-labelled NP by reaction with perylenetetracarboxylic acid monoanhydrides [22], or for the reaction of other activated derivatives of dyes [23].

The observed solvent shift of the fluorescence emission of the NP labelled with MPD and BPD clearly demonstrates that the dye molecules are located at the surface and only in a negligible amount in the interior of the NP [5], although the dye is present in the reaction mixture during synthesis. Pure metallic NP are not labelled by the dyes applied here, as long as there is no oxidized material on the surface. The triethoxysilyl group has no anchor point on a purely metallic surface, and the dye can therefore be bound only losely, by adhesion. Consequently, we did not observe any labelling of pure noble metal NP.

**Figure 3:**
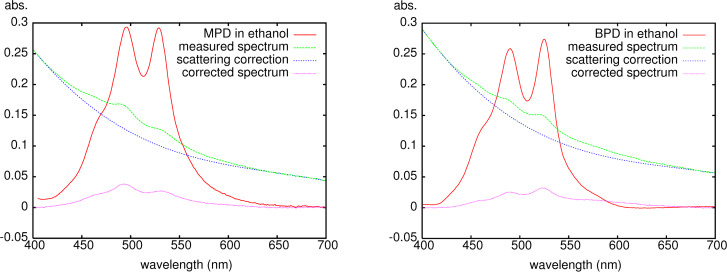
Scattering corrections to the experimental absorption spectra of labelled NP dispersions in ethanol. Left: MPD on the surface of silica NP (average diameter 105 nm, standard deviation 23 nm, 500–700 MPD molecules per particle); right: BPD in the core of silica-coated polyorganosiloxane NP (average diameter 22 nm, standard deviation 5.3 nm; 4.8–5.6 BPD molecules per particle). Absorption spectra of the free perylenediimide dyes MPD and BPD are included for comparison.

It would be desirable to have an estimate of the average number of fluorescent dye molecules attached to a nanoparticle. For low dye loading, one can use stepwise bleaching of single fluorescent molecules on a nanoparticle in a confocal microscope [5]. When the amount of dye molecules at the surface is sufficiently high to be detectable by UV–vis spectroscopy, one can in principle use absorption for photometric determination. The problem with this approach is the strong light scattering by the NP which overlays the absorption. We therefore developed a correction method to remove the scattering fraction from the spectra. This is a difficult task by theoretical means, particularily for polydisperse particles, and we therefore voted for an empirical correction. As Rayleigh scattering is wavelength-dependent with λ^4^ while Mie scattering is less, we reasoned that the scattering contribution to what is the measured “absorbance” could be approximated by a polynome fitted to values of the measured spectrum in regions where no dye absorption is observed. A third-order polynome should have sufficient flexibilty to do the job. As a typical example, we show here how to estimate the covering of silica NP with average diameter 105 ± 23 nm obtained by the Stöber synthesis with MPD (all sizes reported in this review were determined from TEM pictures). First, we determined the molar extinction coefficient of MPD itself in ethanol for the two absorbance maxima at 491 and 524 nm (ε = 39000 l·mol^−1^·cm^−1^ in both cases). Then an absorption spectrum of the labelled NP dispersion was measured in a suitable wavelength range (for perylene dyes, 400 to 700 nm) at a convenient concentration. The range is chosen such that the dye absorbance is negligible at the margins. Two measured data points at each margin are used for the fitting of a third-order polynome used as approximate scattering correction (*C_scat_* = *a*λ^3^ + *b*λ^2^ + *c*λ + *d*, where the coefficients *a* to *d* are determined). This polynome *C_scat_* is then subtracted from the measured spectrum. The resulting curve reflects in principle the contribution of the dye molecules alone. Assuming that the molar extinction coefficient ε for dye molecules bound to the surface of nanoparticles is similar to that of the free molecules, and knowing the number of particles per volume, we can estimate the number of dye molecules per particle. An example for such an estimate is shown in Figure 3 (left). Since these particles are spherical (average diameter of 105 ± 23 nm), and each MPD molecule requires ca. 0.24 nm^2^ of space at the surface [5], we can calculate an average surface covering by dye molecules of 0.3–0.5%. The main error in this estimate is probably due to changes in the molar extinction coefficient upon anchoring at the surface (Figure 3 (left) shows two maxima with different absorbance where free MPD has the same). Since the surface coverage is low, we expected that the labelling would not interfere with biological and medicinal investigations, and this was indeed confirmed in parallel toxicity studies with labelled and unlabelled silica [10–11] and titania [1] NP.

### Silica nanoparticles with a fluorescent polyorganosiloxane core

**Figure 4:**
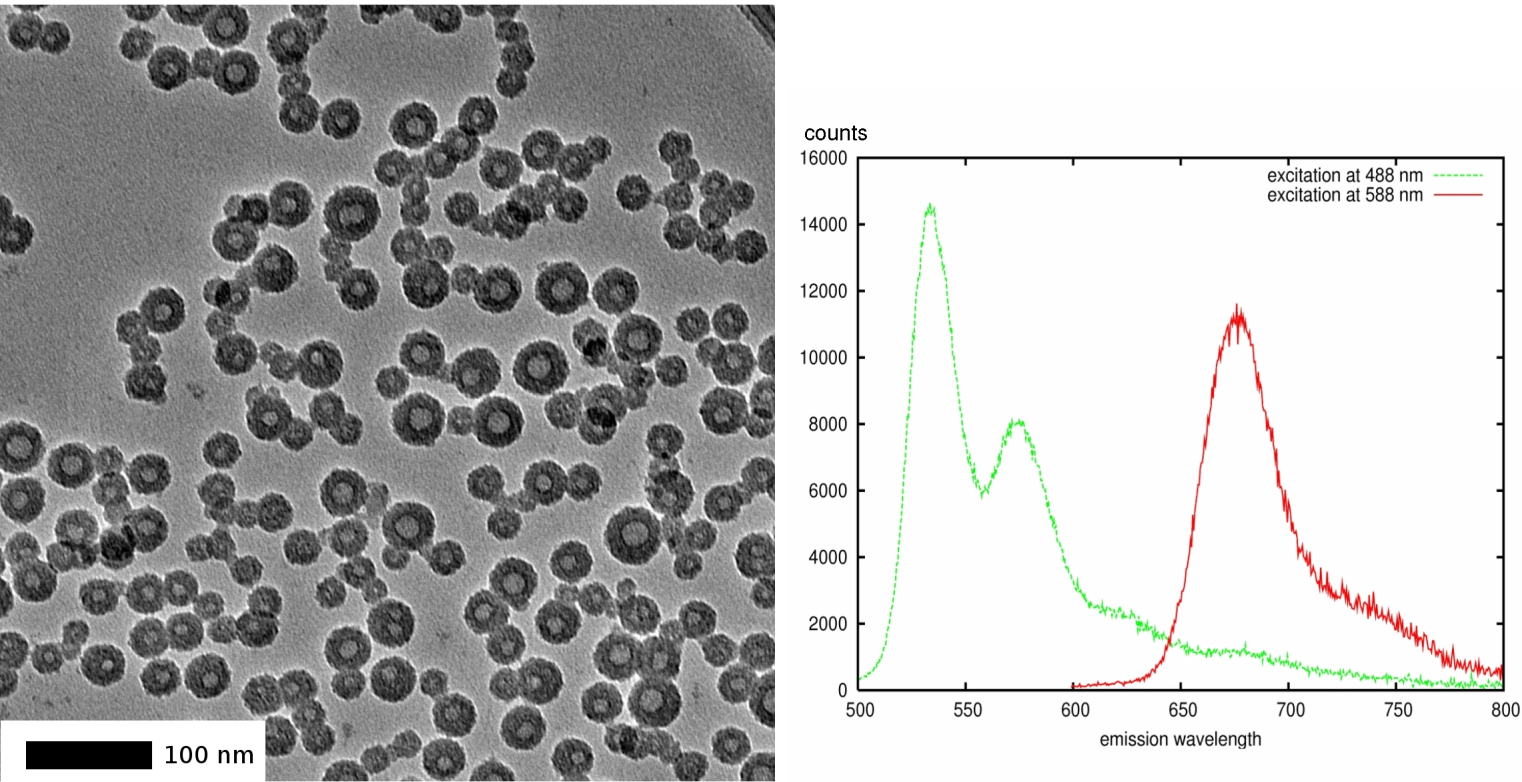
Left: TEM image of core-shell NP with secondary SiO_2_ layer. Average diameter: 31 ± 11 nm, with a core diameter of 11 ± 3 nm. Right: Fluorescence emission spectra of double-labelled core-shell NP in ethanol showing separated BPD and Cy5 fluorescence.

Although the amount of fluorescent dye on the surface of the silica NP described above is very low, it is in principle desirable to avoid any contact of the dye with living cells in biological experiments. This can be achieved by constraining the dye in the core of the NP, isolating it by a shell made from the material to be studied. We therefore investigated whether the perylene-derived BPD dye can be immobilized in a polyorganosiloxane network which in turn could be isolated by a silica shell. This is indeed possible. We modified existing procedures [24–27] for the slow co-hydrolysis of methyltrimethoxysilane and dimethyldiethoxysilane in the presence of BPD in micelles formed by 4-dodecylbenzenesulfonic acid. When the formation of the polyorganosiloxane core incorporating BPD is complete (≈5 d), a first thin silica shell is added by further reaction with tetraethoxysilane (TEOS) during three days. At this stage the NP can be isolated from the reaction mixture [5]. The primary shell can now be enlarged by a secondary shell in a reaction with TEOS under Stöber conditions. The final core-shell NP have total diameters in the range of 30 ± 11–100 ± 25 nm with a fluorescent core of 10 ± 3–30 ± 9 nm. A typical TEM picture is shown in Figure 4 (left). The amount of dye incorporated is, however, limited. It seems that the very large BPD molecules do not easily enter the micelles where the polyorganosiloxane network is formed, but tend to precipitate from the aqueous reaction mixture. The average number of dye molecules in the core can be estimated by the same photometric procedure outlined above. For the symmetric BPD molecule the molar extinction coefficients are lower than for MPD (ε = 2200 l·mol^−1^·cm^−1^ at 522 nm and ε = 2080 l·mol^−1^·cm^−1^ at 488 nm in ethanol). Figure 3 (right) shows a typical example of the procedure. We used here NP with an average diameter of 22 ± 5.3 nm (no secondary shell added) and obtain an estimate of 4.8–5.6 dye molecules per core. Neglecting the primary shell (which is not detectable by TEM) and taking the whole particle as core, we can calculate that the dye occupies only 0.1% of the particle volume. This means that the individual molecules are reasonably separated from each other without the danger of self-quenching of the fluorescence.

During the process of formation of the primary shell one can easily modify the surface without influencing the fluorescent core. Thus, we introduced thiol (–SH) groups at or near the surface by replacing a part of the TEOS used for the formation of the primary shell by (3-mercaptopropyl)triethoxysilane (MPS, up to 15 mol %). The number of thiol groups accessible by other chemical reagents could be determined by a modification of the photometric procedure using Ellman’s reagent, 5,5′-dithiobis(2-nitrobenzoic acid). The standard conditions for the determination of thiol groups, e.g., in molecules or proteins involve aqueous solutions and pH control [28]. This was, however, not applicable to the dispersions of silica NP because of increased agglomeration. We therefore performed the reaction in dry ethanol and ensured the quantitative formation of the yellow dianion of 5-thio-2-nitrobenzoic acid (maximum absorbance at 421 nm) by addition of a large excess of (3-aminopropyl)triethoxysilane (APS). Under these conditions, the NP sediment during the first hour, and the maximum colour is observed after ca. 150 min and can easily be measured without interference of scattering effects. In case of incomplete sedimentation the solution can be centrifuged after the maximum colour has developed and the supernatant solution used for the photometric determination. For calibrating we used free MPS (maximum absorbance after 15 min) and obtained a straight calibration line which is valid up to *abs* = 1.1. For NP prepared by replacement of 10% of TEOS by MPS for the formation of the primary shell (average diameter 22 nm, standard deviation 5 nm) we determined a total of 1090 thiol groups per nanoparticle. Assuming equidistant distribution at the surface we can calculate an average distance of ca. 1.2 nm between two neighbouring thiol groups.

The new functionality can be used for further modifications. We demonstrated this by introducing the quenchable dye Cy5.5 (in contrast to the non-quenchable perylene dyes) by reaction with its NHS ester in ethanol for one day. The dye is connected to the sulfur by a thioester link which is stable in aprotic solvents but undergoes slow hydrolysis in water or transesterification in ethanol. Nevertheless the NP labelled with two colours show the reasonably separated fluorescence of the perylene dye (excitation 488 nm, emission maximum 533 nm) from Cy5.5 (excitation 588 nm, emission maximum at 675 nm) (Figure 4, right).

Since the labelling procedure for the core is experimentally convenient (although time-consuming) we tried to extend it to other APS-connected fluorescent dyes, but failed completely. No fluorescence was detected in the polyorganosiloxane core when we used APS-modified acridine orange, Cy3, Cy5, ATTO 488, ATTO 590 or ATTO 647N. There was no improvement when we replaced APS with bis(3-triethoxysilylpropyl)amine to obtain derivatives with two −Si(OEt)_3_ groups. A common feature of all dyes which failed is their cationic nature. We therefore think that they have difficulties to pass through the membrane of the micelles formed from (easily deprotonated) 4-dodecylbenzenesulfonic acid.

### Ceria nanoparticles

Ceria (CeO_2_) NP do not occur in nature but are man-made. Their principal application is for catalysis. Due to the easy change of the oxidation state (Ce(III) and Ce(IV)) they can act as redox catalysts themselves, e.g., for the regulation of combustion and many other applications [29–30]. Even more important is their use as carrier material for metallic catalysts. For automobile catalytic converters meso- or nanoporous ceria or alumina is used as washcoat (on which the noble metal catalysts are deposited) for cordierite, the latter supplying the required mechanical strength. Here the oxygen storage capacity of CeO_2_ improves the performance of the noble metal catalysts. Despite the impressive progress in the reduction of harmful gases (CO, NO_x_ etc.) and carbon black in the exhaust gases one has to be careful not to overlook potential side effects. Due to the high thermal and mechanical stress the converter materials are not indefinitely stable but decompose, leading to airborne particles (nano- to micrometer scale) containing mainly the washcoat and the catalytically active metal, which come into the environment and finally into living organisms like humans [31]. We may call this an unintended exposure to unintended particles. To study the biological effects of such particles was an important topic on the agenda of the NPBIOMEM research cluster.

It is not very feasible to extract these NP from the environment due to their low concentration in mixture with other materials of similar size; only the overall noble metal content of sediment samples [32] or of exhaust gas [33] and air in cities [34] has been determined. Therefore a practical synthesis of model particles with controlled composition, morphology and size was required. For palladium on alumina [35] and platinum on alumina [36] or alumosilicate [37] this has been achieved. We set out to prepare model NP of CeO_2_ decorated with the catalytically important noble metals platinum, palladium and rhodium. As for the silica particles, fluorescence labelling is essential for the biophysical studies.

#### Controlling size and shape of ceria nanoparticles

**Figure 5:**
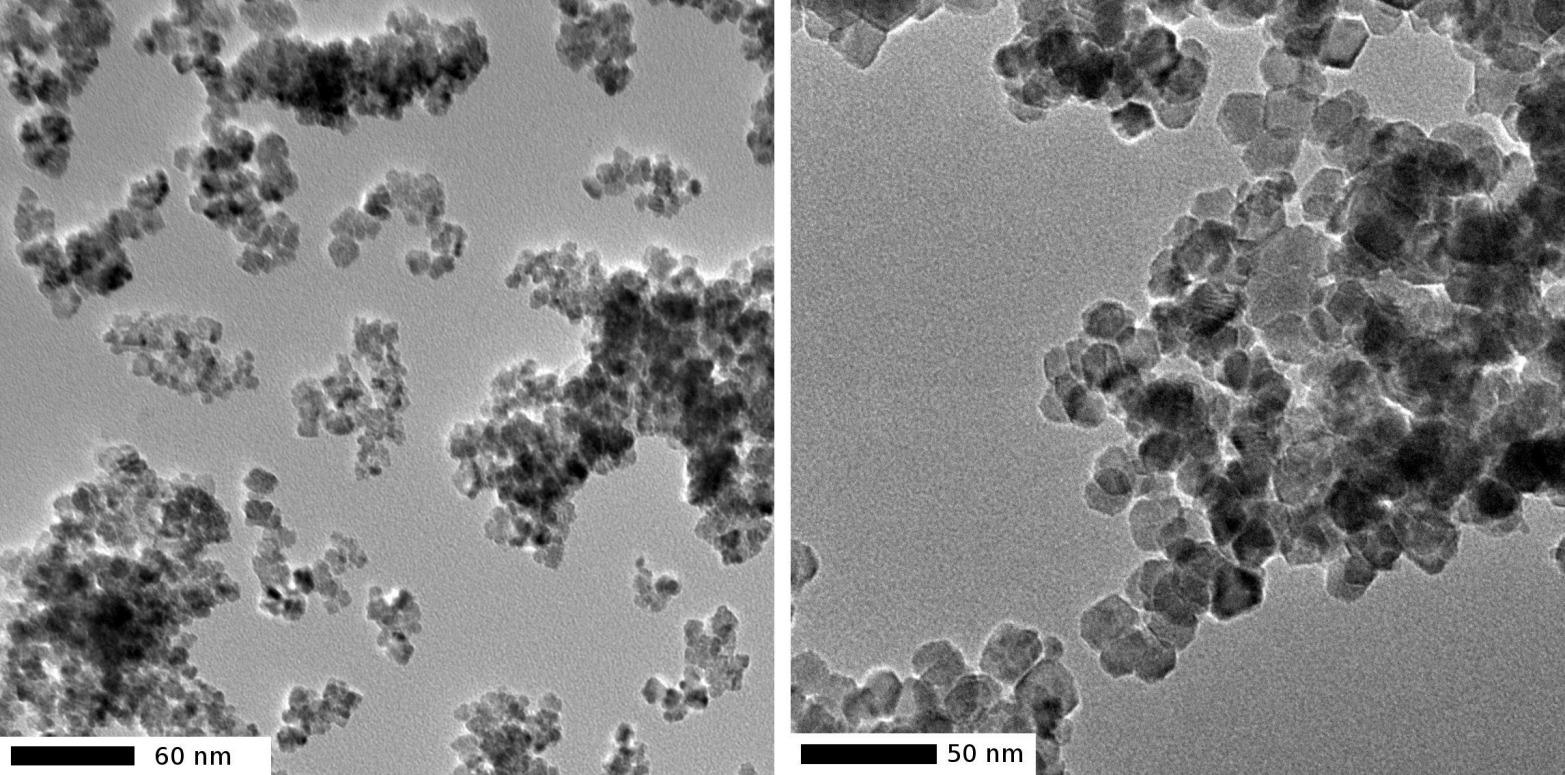
TEM images of CeO_2_ NP prepared according to [43]. Left: solvent ethanol/water 4:1, spherical particles, average diameter 8 ± 2 nm; right: solvent water, elliptical and octahedral particles, circumscribed sphere diameter 35 ± 10 nm.

Many commercial ceria NP have a broad size distribution and widely varying shapes. Their direct labelling with the dye-APS conjugates in analogy to what is described for silica (see above) is possible but the particles are not suitable for the planned biological investigations. We therefore checked several literature procedures for the synthesis of ceria NP [38–41] and found the procedure with air oxidation of cerium(III) nitrate in ethanol/water mixtures in the presence of ammonia very convenient [42–43]. At 60–70 °C (open flask) one obtains small crystalline NP in ethanol/water 4:1 as solvent of almost spherical shape (average diameter 8 ± 2 nm) and a mixture of elliptical and octahedral (or intermediate) shapes in water (average circumscribed sphere diameter 35 ± 10 nm). For a detailed discussion of these shapes, see [44]. The NP have a strong tendency towards agglomeration with no possibility to control the size of the agglomerates; they vary widely just as the sizes of the particles from exhaust gas do. Typical TEM images are shown in Figure 5. We tried to make these NP even more similar to the airborne decomposition products of automobile catalysts by annealing at 400 °C for four hours, because an evaluation of toxicity studies on CeO_2_ NP has revealed that their physiological properties strongly depend on the preparation methods. While low temperature particles seem to have mostly beneficial effects on cells by reducing the amount of reactive oxidizing species (ROS), the contrary was found for high temperature particles (as for the true automobile catalyst decomposition products) which increase the amount of ROS in cells [45]. However the annealing process dramatically increased the degree of agglomeration, and redispersion in ethanol or water became difficult, as well as attempts to label them with ATTO 647N-APS. They rather behave like the particles of several micrometers diameter which are in the upper range of the emitted catalyst decomposititon products. Since such big particles are hardly internalized by cells, one would not expect them to have much interaction with biological systems. Therefore, the original non-annealed NP were used for biomedical [46] and uptake studies [47], both articles in this issue.

What cannot be avoided must be controlled. Since agglomeration is a question of surface properties, it should be possible to control it by surface-active reagents. Consequently, we tried to stabilize the NP with surfactants like IGEPAL CO-520 but with no appreciable success. The only efficient approach up to now to controlled agglomeration of ceria NP is their synthesis from cerium(III) nitrate in ethanol/water mixtures in the presence of polyvinylpyrrolidone (PVP), at temperatures exceeding 120 °C [48]. Under these conditions, nitrate acts as oxidizing agent, and the reaction can be done in closed vessels. The overall reaction is 3Ce^3+^ + NO_3_^−^ + 4H_2_O → 3CeO_2_ + NO + 8H^+^.

The authors suggest that PVP interacts with the crystal seeds and prevents an increase in size over the limit of 8–10 nm. These particles then agglomerate by merging their PVP coverings until a maximum size under the reacion conditions is reached. Upon prolonged reaction the particles may undergo ruptures and decrease their size again. This mechanism has been suggested ad hoc from the observation of size and shape (roughness) during the reaction.

**Figure 6:**
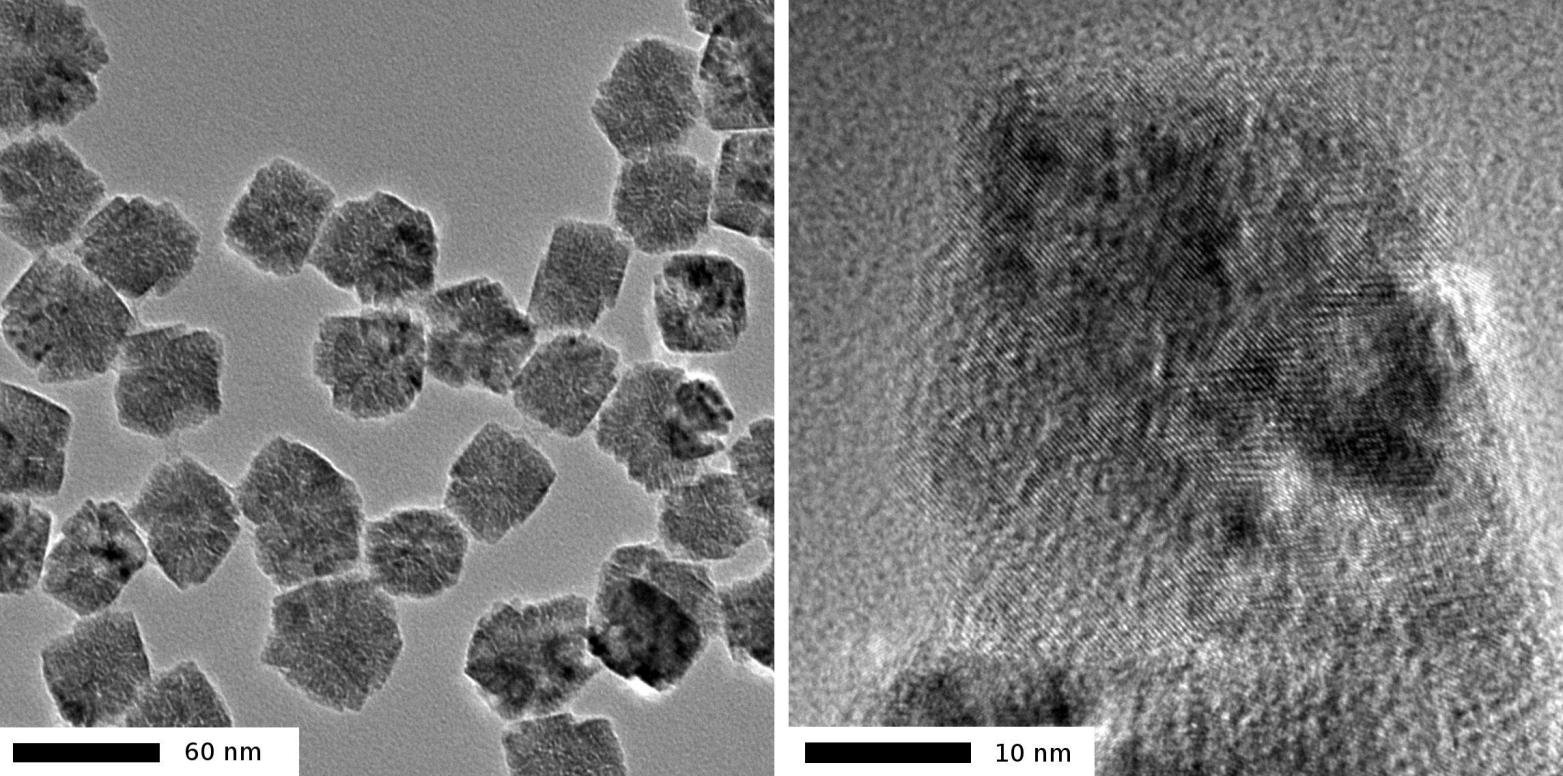
TEM images of agglomerates of CeO_2_ NP prepared according to [48], with average diameter of the circumscribed sphere 51 nm (standard deviation 4 nm).

The agglomerates are easily purified by removing PVP by centrifugation and washing with water several times. The final particles are best stored in ethanol where they are stable for some months. The shape of the agglomerates varies between spherical and octahedral, and their size can be determined from TEM images by measuring the diameter of the circumscribed sphere. Up to now we were able to obtain average particle sizes between 39 ± 4 and 260 ± 40 nm. Typical images are shown in Figure 6. The HRTEM image (right) shows that the large particle is indeed composed of 8–10 nm size NP.

There are several parameters which define the size of the agglomerates. The influence of reaction time and the ethanol/water ratio was already studied in detail in [48]. We could confirm that the size increases with time, reaches a maximum after ca. 15 hours (the precise time depending on the other reaction conditions), and then starts to decrease slowly. Higher ethanol/water ratios lead to smaller final agglomerates. Below 2:1 the size distribution starts to broaden, and in pure water, the agglomerates are no longer monodisperse but a mixture of the expected large (>200 nm) and rather small (<100 nm) particles. We found the best homogenity of the particle size at a ratio of 3:1 and used it for all further syntheses. The reaction temperature is of minor importance; the oxidation of Ce(III) by nitrate starts at ca. 120 °C, and the size only slightly increases when going up to ca. 170 °C. The ratio of PVP to Ce(NO_3_)_3_·6H_2_O is certainly the most important parameter, the higher it is the smaller the particles. As a rule of thumb one could say that increasing this ratio by a factor of 8 will result in a reduction of the particle size by a factor of 4. In contrast, the absolute concentration of the two components is less important; as long as PVP is still soluble in the solvent mixture, there is no appreciable change in size provided that the PVP/Ce(III) nitrate ratio remains constant. The monodisperse agglomerates are ideal starting materials for the synthesis of metal-decorated models for the decomposition products of automobile catalytic converters.

Small agglomerates (average diameter below ca. 100 nm) have an intrinsic tendency to further agglomeration; “super-agglomerates” with several micrometers size (where the original agglomerates can still be distinguished in the TEM images) are occasionally observed in the reaction products. Since they occur in strongly varying amounts we cannot give a typical percentage for the degree of aggregation. They can be removed almost completely by centrifugation at low gravity from ethanol dispersions and may be of interest as models for the larger particles from the catalyst decomposition. Attempts to de-agglomerate these super-agglomerates in order to obtain the original small particles by ultrasound treatment were not succesful.

#### Ceria nanoparticles decorated with noble metals

Deposition of finely dispersed noble metals on porous materials is a common technique for the preparation of heterogeneous catalysts. It generally involves impregnation of the carrier with a soluble precursor and calcination, or precipitation and reduction followed in most cases by calcination which is expected to increase the fixation of the metal on the carrier. With respect to (meso)porous ceria, this has been described for, e.g., rhodium [49–50] (impregnation), platinum [51] (ultrasound-assisted reduction), and gold [52–53] (impregnation and photochemical reduction, respectively). In principle, these procedures should be applicable to ceria NP as well, but in the light of the observed increase in agglomeration after calcination of ceria NP (see above) some caution is justified when the preparation calls for this technique. Several approaches to noble-metal-decorated ceria NP have been suggested recently: for gold and palladium by reduction [48,54–55], and for gold and platinum by microwave-assisted reduction [56]. In order to prevent agglomeration during calcination of Pt/CeO_2_ hybrid particles, a procedure was reported that adds H_2_PtCl_6_ to ceria NP, followed by gel/sol formation with TEOS, calcination and finally removal of the SiO_2_ protective shell with NaOH [57]. With our ceria NP this failed because of extensive platinum nucleation in the gel, the resulting platinum NP not being attached to CeO_2_. Photochemical attachment of platinum NP to silica core–shell (described above) and ceria NP also failed for the same reason (K_2_PtCl_4_ solution, citrate buffer, xenon lamp). A procedure based on the idea of electrostatic attraction between NP finally worked for our systems. The CeO_2_ NP after coordination with 6-aminohexanoic acid (AHA) obtain a strongly positive ζ-potential. Platinum NP stabilized by PVP, in contrast, develope a negative ζ-potential. Forming the platinum NP by reduction of K_2_PtCl_4_ with borohydride in the presence of PVP and AHA-stabilized ceria NP should therefore lead to a better connection of both [58]. Having possible simplifications of the experimental procedure in mind, we checked if one could omit AHA and PVP; in this case the ζ-potentials of the particles have lower absolute values but maintain their sign [58]. What still encouraged us was an analogous reduction of PdCl_2_ with borohydride in the presence of ceria NP without additives that resulted in a reasonable palladium load [55]. It turned out that the simplified procedure gave the same particles as the joint addition of AHA and PVP. Thus, our final decoration procedure consists in mixing a dispersion of the ceria NP in water with the noble metal precursor (K_2_PtCl_4_, PdCl_2_, RhCl_3_) and stirring between room temperature and 70 °C for ca. 20 h. The long reaction time is necessary to allow for attachment of the precursor to the ceria NP by slow ligand exchange at the noble metal, replacing Cl^−^ with the hydroxy groups at the surface, which results in the formation of nucleation seeds directly at ceria. Reduction with KBH_4_ leads to the desired decorated ceria NP, and workup consists simply in several centrifugation/redispersion steps in water and ethanol. The particles are readily labelled with ATTO 647N-APS in ethanol (120 °C, 4 h). It is not possible to label the ceria NP first and decorate them afterwards as the dye is oxidatively destroyed by Pt(II). Detailed synthetic procedures will be described elsewhere.

**Figure 7:**
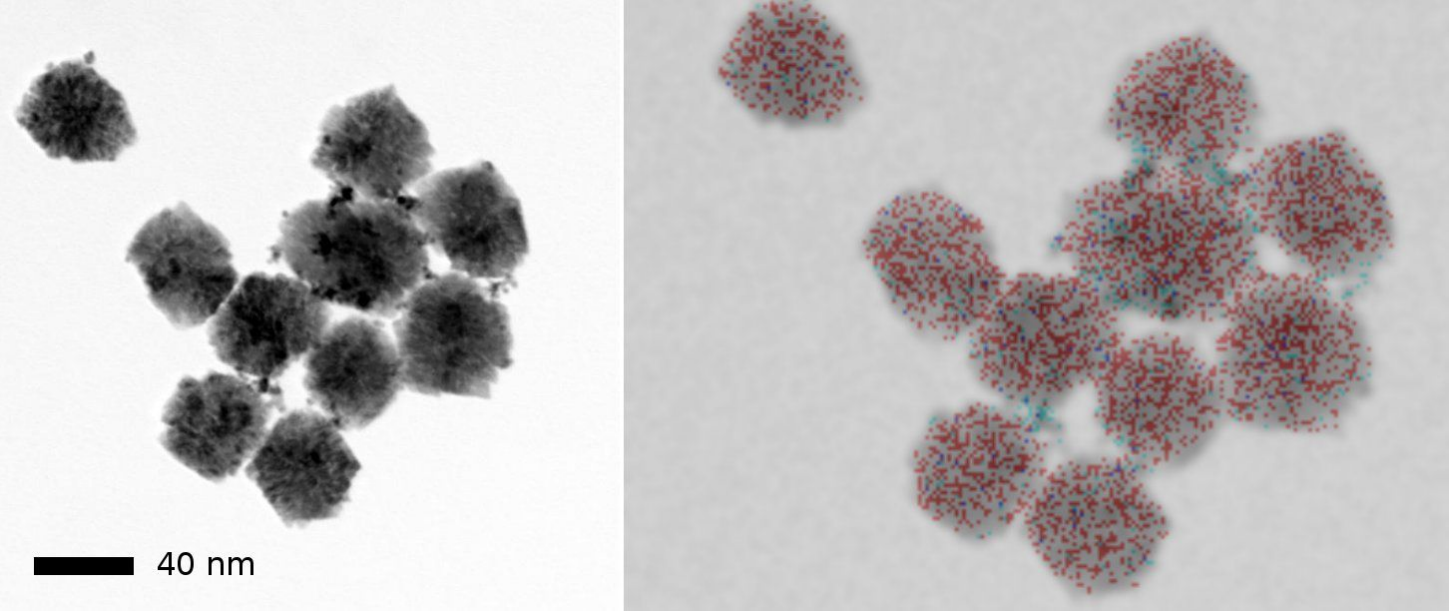
STEM image (left) and EDX mapping (right) of agglomerates of Pt-decorated CeO_2_ NP (diameter of the circumscribed sphere: 44 ± 4 nm). In the overlay EDX image, Ce is shown in red and Pt in blue.

**Figure 8:**
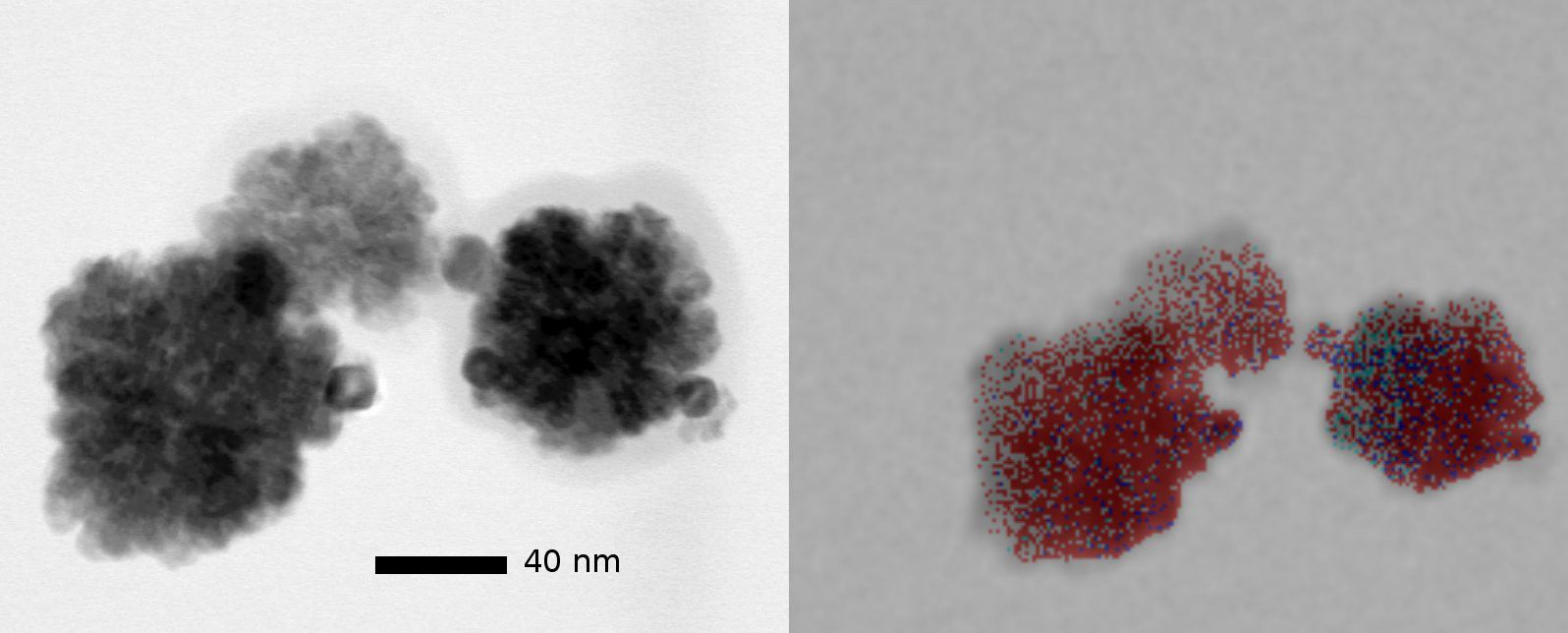
STEM image (left) and EDX mapping (right) of agglomerates of Pd-decorated CeO_2_ NP (diameter of the circumscribed sphere: 102 ± 10 nm). In the overlay EDX image, Ce is shown in red and Pd in blue.

The decorated ceria NP were analyzed by TEM and EDX for size and noble metal distribution. A typical result for controlled agglomerates is shown in Figure 7. The STEM picture (left) shows platinum as dark spots while cerium as the bulk material has a lower contrast. The EDX mapping confirms the colocalization of platinum and cerium. The noble metal does not form a continuous layer at the surface of ceria but is concentrated in NP of 2–5 nm diameter. A comparison of the EDX intensinty of cerium and platinum suggests that approximately 10% are due to platinum. This corresponds to the maximum load of platinum on the ceria NP that can be obtained without admixture of non-attached platinum NP. Since the platinum NP are larger than the space available between the ceria NP from which the agglomerates are formed, we can speak of a true “decoration” of the surface. If the surface of an agglomerate of 50 nm diameter would be totally covered with platinum NP of 2 nm diameter, 37% of the total weight of the hybrid Pt/CeO_2_ particle would consist of platinum. In practice, we could not apply more than 8% of platinum without provoking nucleation of platinum NP not attached to ceria. Similar observations were made with palladium and rhodium. In the STEM images, however, the contrast difference between cerium and the metal is considerably lower, and EDX mapping is the best way to determine the extent and the distribution of decoration. Figure 8 shows an example for palladium. Local metal concentrations on the right particle can be detected, but the lower resolution of EDX compared with TEM imaging does not allow for more precise localization.

The decorated particles can be stored in ethanol for some weeks, but they may undergo changes after some months. We observed the formation of super-agglomerates, together with wires and sheets of crystalline CeO_2_ in the micrometer range, obviously formed by recrystallization, but not in all samples. The presence of traces of water in the solvent may be the reason for this instability; a related observation is the formation of ceria nanosheets when cerium(III) nitrate was added to an aqueous reaction mixture over several hours, whereas NP were formed when the addition was rapid [59]. Although the platinum metal NP contained in the samples are seemingly unchanged although detached from ceria, they probably are part of the recrystallization process, since this behaviour has not been observed for pure CeO_2_ NP and agglomerates. Attempts to stabilize the decorated NP by annealing at 400 °C were only partially successful. Increased agglomeration of the ceria part was observed in some cases, but even more frequently we found a considerable increase in the size of the noble metal particles, particularily for palladium. Annealing is therefore not the method of choice. It is rather advisable to dry the particles carefully and store them in totally dry ethanol.

In the final particles used for the biophysical investigations, noble metal NP at the surface of ceria have the ATTO 647N fluorescence label in close vicinity. One might expect electronic interactions between both which may result either in fluorescence quenching or in enhancement, depending on the distance, orientation and environment of both components [60]. Fluorescence lifetime imaging microscopy (FLIM) on CeO_2_ agglomerates (50 ± 5 nm diameter of the circumscribed sphere) with and without platinum decoration did not show any difference between both samples. We can therefore conclude that there is no efficient energy transfer between metal and fluorescence dye.

## Conclusion

We have reviewed the preparation and characterization of particles having a fluorescent polyorganosiloxane core containing perylenediimide dye and a silica shell which can be further modified with reactive thiol groups. The number of thiol groups per nanoparticle can be determined by a modification of the Ellman’s photometric procedure. We have also developed a photometric method to estimate the amount of dye molecules attached to a nanoparticle by subtracting scattering effects form the absorption spectra. In the second part we have reviewed our approach to noble metal decorated ceria NP which serve as models for the particles formed by mechanical decomposition of automobile catalytic converters. We succeeded in controlling the agglomeration of small (8–10 nm) ceria NP and obtaining almost monodisperse agglomerates (40–260 nm) which can be decorated with noble metal NP (Pt, Pd, Rh, 2–5 nm) by reduction of suitable precursors. Fluorescence labelling with ATTO 647N leads to the automobile catalyst decomposition model particles whose biophysical properties are now studied. Gold-decorated ceria nanoparticles have a surprisingly high efficiency for reducing the amount of reactive oxygen species (ROS) in living cell lines and thus a beneficial effect [61]. If platinum group metals-decorated ceria NP do the same or even more is under investigation.
